# The utilisation of international consensus definitions of the disease polytrauma: a systematic review

**DOI:** 10.1007/s00068-026-03339-6

**Published:** 2026-09-29

**Authors:** See Yoon Kim, Zhengchao Xu, Zsolt J. Balogh

**Affiliations:** 1 Department of Traumatology, John Hunter Hospital, Newcastle, NSW Australia; 2 Discipline of Surgery, School of Medicine and Public Health, University of Newcastle, Newcastle, NSW Australia; 3 Injury and Trauma Research Program, Hunter Medical Research Institute, Newcastle, NSW Australia

**Keywords:** Trauma, Polytrauma, Trauma centre, Trauma system

## Abstract

**Purpose:**

The disease of polytrauma is associated with significant healthcare burden and poor prognosis. The validated Newcastle and the Berlin consensus definitions of polytrauma have been proposed more than a decade ago for standardising clinical, scientific and advocacy related communications. This systematic review aims to evaluate the uptake of the Newcastle and the Berlin definitions of polytrauma since their introduction.

**Methods:**

This systematic review (PROSPERO: CRD420251119950) adhered to the Preferred Reporting Items for Systematic Reviews and Meta-Analyses (PRISMA) guidelines. A literature search of original research studies reported in English on polytrauma patients from 1st January 2015 to 31st of December 2025 was conducted, using Medline, Embase, Scopus, Pubmed, CINAHL and CENTRAL. Specific focus was on the utilisation of the Berlin and the Newcastle definitions, but all explicitly stated definitions of polytrauma were analysed.

**Results:**

A total of 2354 studies were identified through initial literature search. 1491 studies were excluded after screening by title and abstract. The full text review of 863 studies was performed and resulted in 88 eligible studies after the exclusion of 732 studies with no explicit definitions of polytrauma and 43 studies on non-human subjects. Ten studies (11.4%) used the Berlin definition of polytrauma, and six studies (6.8%) reported using the Newcastle definition. The consensus definitions were reported the most in 2024, with 2 studies using Newcastle and 3 studies using the Berlin definition of polytrauma.

**Conclusions:**

Despite the establishment and utility in accurately representing the polytrauma population, the Newcastle and the Berlin definitions of polytrauma remain under-utilised in the current literature.

## Introduction

The term polytrauma has historically been used in both clinical and academic contexts to describe injuries involving multiple body regions physiological compromised and systemic inflammation. Lack of an objective and reproducible definition has led to significant variability in its usage, often being applied interchangeably to describe severely injured or critically ill patients with multiple traumatic injuries [1, 2].

The Injury Severity Score (ISS), which is based on the Abbreviated Injury Scale (AIS), is the most frequently used anatomical trauma scoring system. Specific cut-offs in ISS are used to define severe injuries, and is used in referring to multi-trauma, major trauma and polytrauma [3–7]. Most mortality focused international studies define severely injured patients as ISS > 15 [8], while in Australasia is ISS > 12 [9]. However, barytrauma or isolated single system trauma can also meet this criteria, and hence, reporting on based on an ISS cut-offs does not differentiate between a severe single system injury and a polytraumatised patient [4].

In response to these limitations, the Newcastle definition was proposed and validated. It defines polytrauma as the presence of AIS > 2 injuries in at least two body regions [10]. This objective definition was validated by a prospective study at a single trauma centre and by a large European trauma registry [10, 88]. Using these definitions has the advantage of being accessible in retrospect, from any institution or registry that incorporated the AIS for injury coding to calculate ISS. Polytrauma defined by the Newcastle definition consistently demonstrated the presence of physiological compromise, systemic inflammatory response [11], high risk of multiple organ failure, and a high mortality rate of 14% [4], which are all descriptive hallmarks of polytrauma. Although it is difficult to define retrospectively from chart reviews, polytrauma patients defined by the Newcastle definition developed systemic inflammatory response syndrome (SIRS) in 92% of the cases [11]. The predictive performance of the Newcastle score reinforces that with right cut-off, polytrauma can be defined accurately without the inclusion of physiological parameters.

The Berlin Polytrauma Definition (BPD) was further developed with the intention to identify the most severely injured polytrauma patients with mortality around 30% in year 2010 standards. This definition is based on the Newcastle definition of AIS > 2 in at least two AIS body regions, added with physiological risk factors (PRFs) hypotension, acidosis, coagulopathy, age and Glasgow Coma Scale (GCS) with specific cut-offs [12, 13]. These additional physiological criteria confirms the value of the Berlin Polytrauma Definition in identifying the most critical polytrauma.

There is an increasing recognition of polytrauma as a complex acute onset chronic disease in response to kinetic energy transfer to the body [14]. This conceptualisation is reflected by the movement towards recognising it in the 11th version of the International Classification of Diseases (ICD) by the World Health Organization (WHO). In this proposal polytrauma will be coded based on objective AIS based definition, which necessitates unified approach globally.

This systematic review evaluates usage of the Newcastle and Berlin definitions of polytrauma in contemporary literature, to identify what proportion of studies mentioning “polytrauma” defined the disease objectively and what proportion was based on validated consensus definition.

## Methods

### Study design

A systematic review of the literature was performed and reported in accordance with the Preferred reporting Items for Systematic reviews and Meta-Analyses (PRISMA) 2020 checklist. The protocol was registered in PROSPERO (CRD420251119950).

### Literature search

A literature search on all original human clinical studies on polytrauma population, published since the establishment of the Berlin Polytrauma Definition, from 1st January to 5th October 2025 (to be further extended), was conducted by the first and second authors using Medline, Embase, Scopus, Pubmed, Cumulative Index to Nursing and Allied Health Literature (CINAHL) and Cochrane Controller Register of Trials (CENTRAL). The search terms, “polytrauma”, “trauma”, “multi-trauma” and “major trauma”, were used.

### Study selection

Studies without stated definition of polytrauma in their methodology were excluded. Patient-reported outcome measure studies were not excluded by design in our current review as outcome type was not a selection criterion. Animal studies, simulation-based studies, reviews, case reports, abstracts, commentaries, theses, proceedings, conferences, editorials, and books were excluded.

### Data extraction

**Fig. 1:**
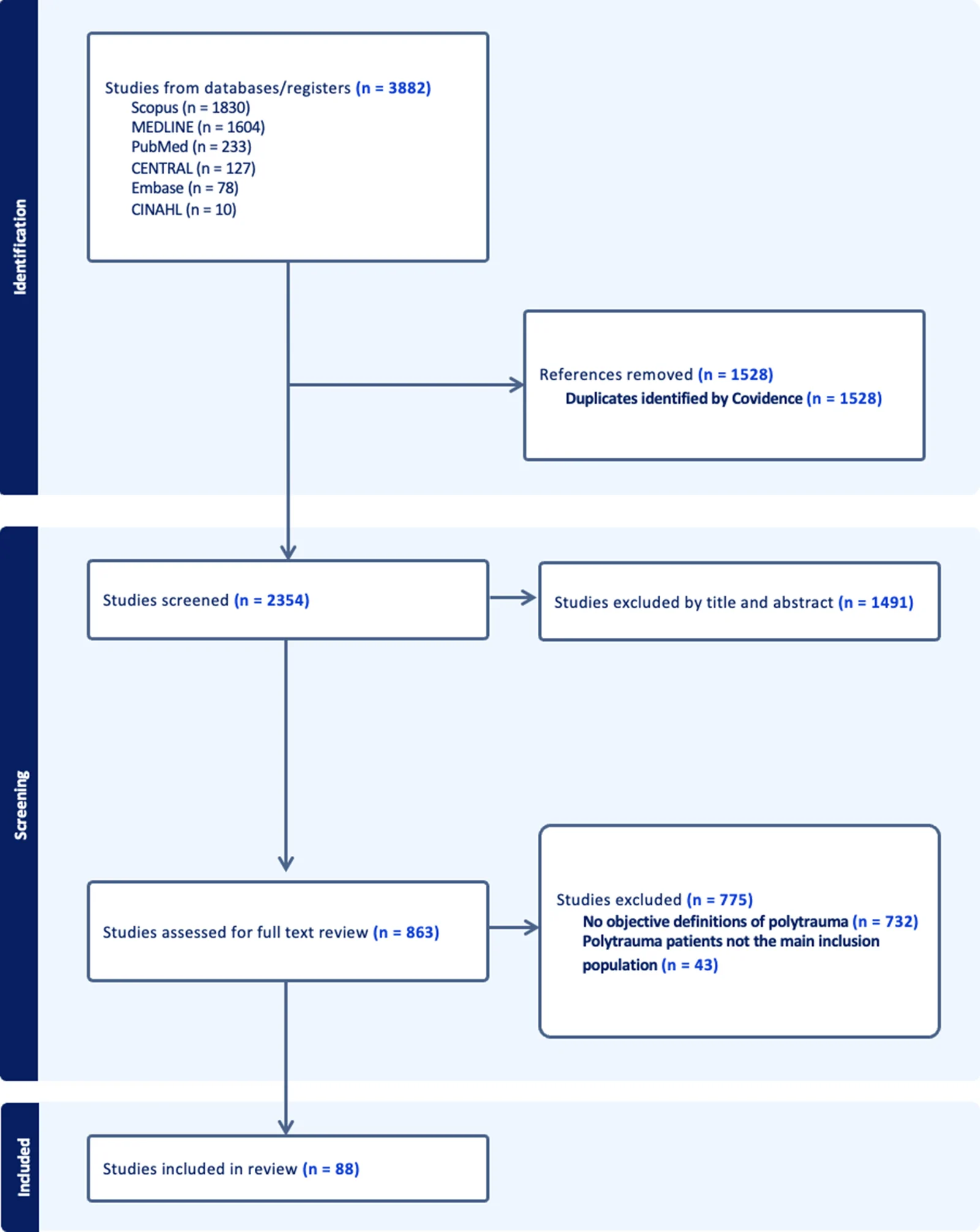
PRISMA flow diagram

Using Covidence, (Covidence systematic review software, Veritas Health innovation, Melbourne, Australia. Available at www.covidence.org), duplicates were automatically removed after the initial identification of studies. The studies were initially screened by title and abstract by two authors and conflicts in decision to include were resolved by the third senior author, with relevant content expertise. The same process was repeated on the papers selected for full text review. All eligible studies were extracted and evaluated for the polytrauma definition utilised. Those using the Newcastle or the Berlin definition of polytrauma were analysed based on year and location of publication. The literature search process is summarised using PRISMA flowchart outlined in Fig. 1.

### Risk of bias assessment and study quality

**Fig. 2:**
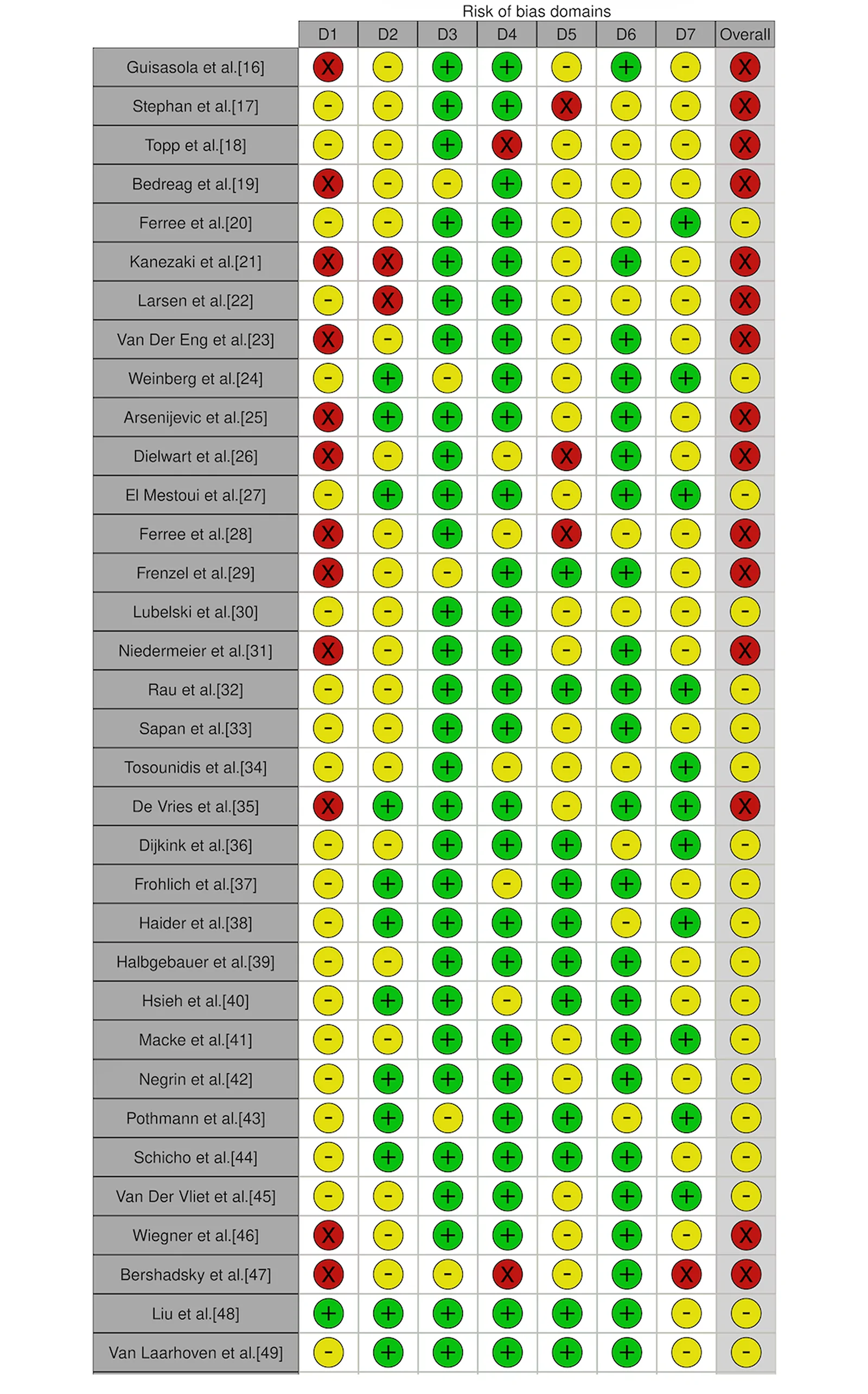

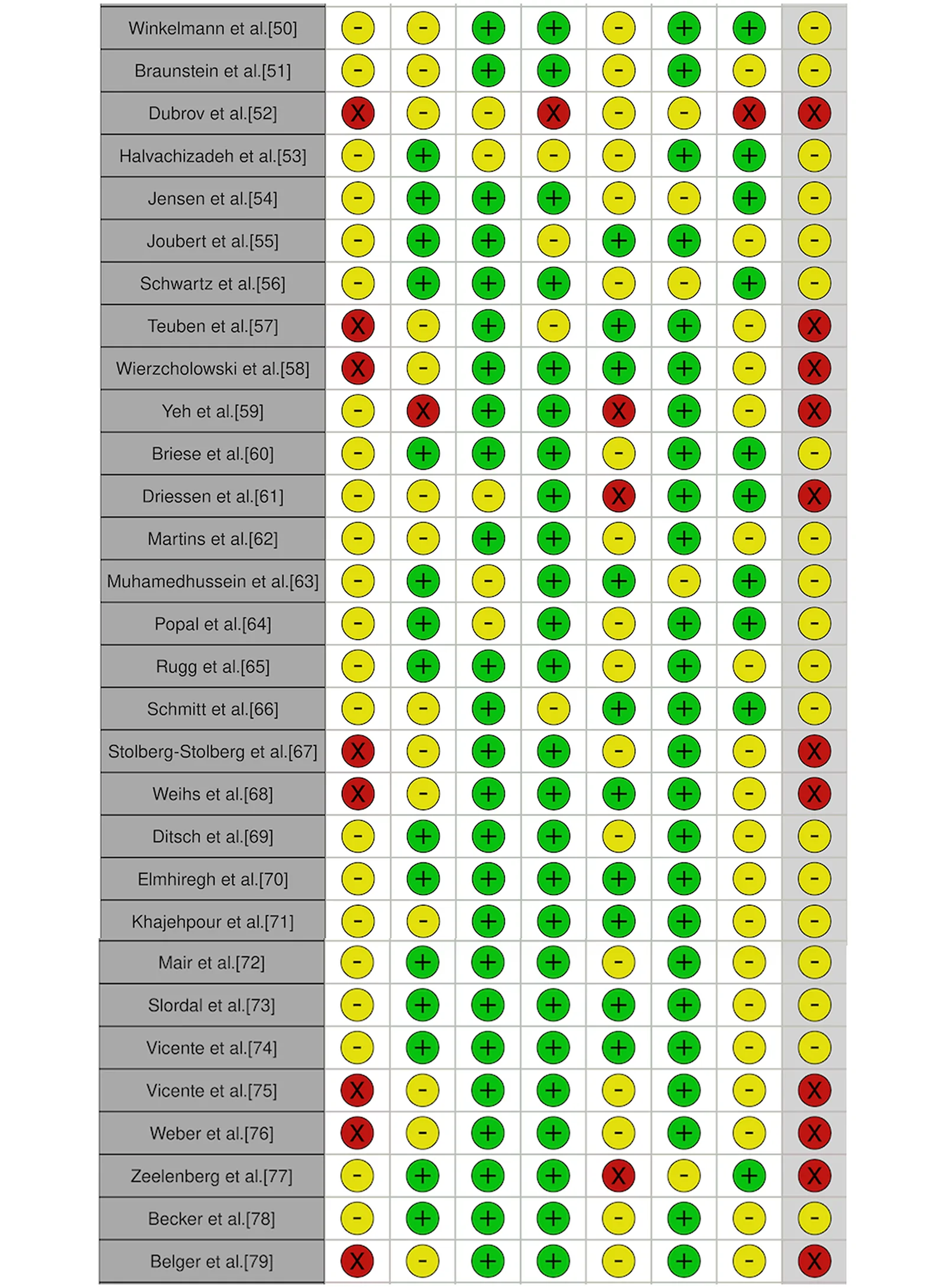

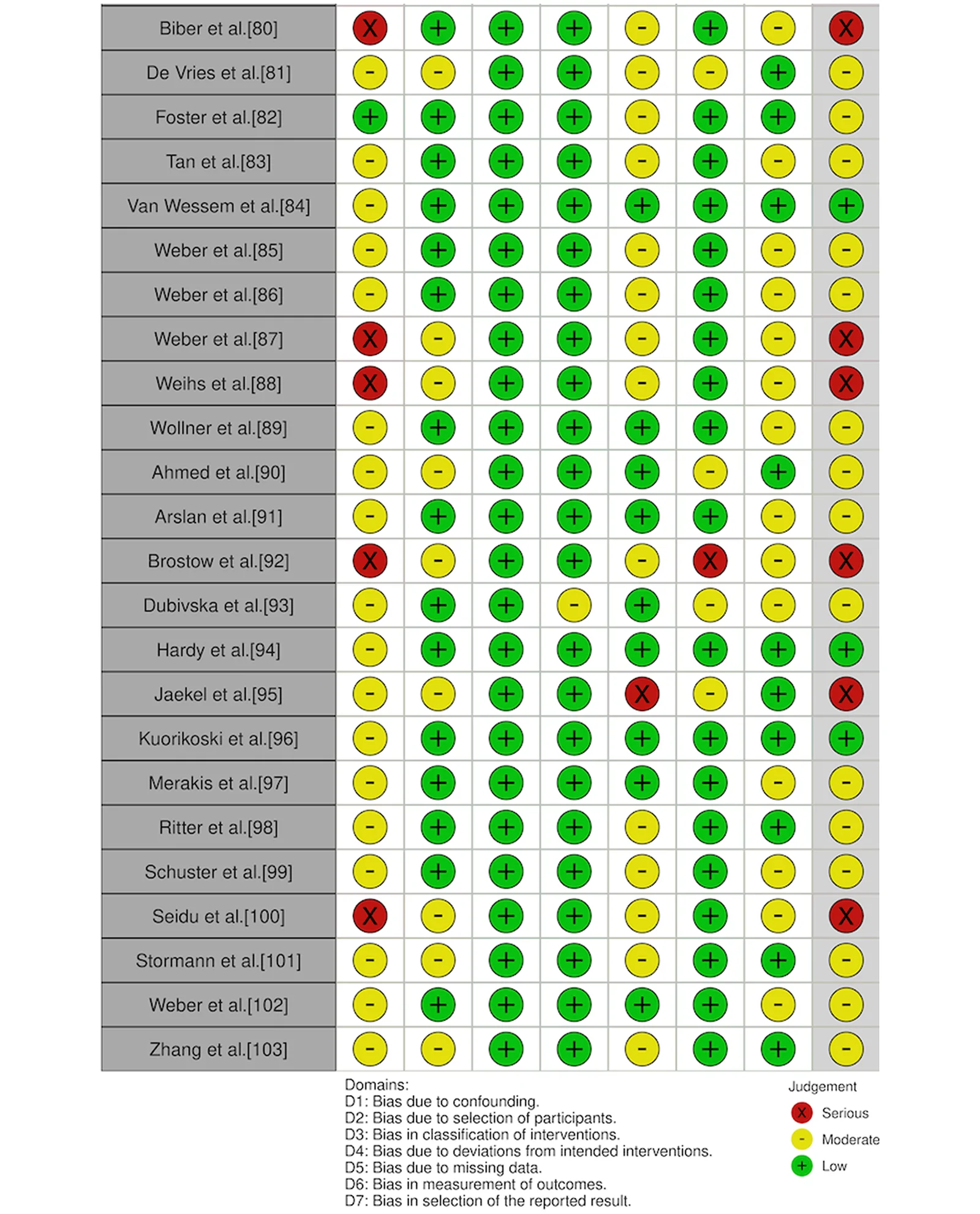
Risk of bias assessment using ROBINS-I

Risk of Bias in Non-Randomised Studies – of Interventions (ROBINS-I) tool was used to assess the risk of bias for the included studies. The results are presented using a traffic-line plot in Fig. 2.

## Results

3882 studies were identified after initial search of electronic databases. Using Covidence, 1528 duplicates were automatically identified and removed. From remaining 2354 studies, 1491 studies were excluded after screening by title and abstract. 863 studies were eligible for full-text review. From these studies, 732 were excluded as no objective definitions of polytrauma were provided and 43 were excluded due to not having polytrauma population as the main inclusion criterion. A total of 88 studies with polytrauma population and with objective definitions of polytrauma were included for review (Table 1). Due to heterogeneity in the study design, meta-analysis was not feasible and was not considered.

88 studies were retrieved in total: 32 prospective (36.4%), 49 retrospective (55.7), 5 cross-sectional (5.7%) and 2 randomised controlled trials (2.3%). Of these, 60 were from Europe (68.2%), 12 from Asia (13.6%), 9 from North America (10.2%), 3 from Africa (3.4%), 2 from Oceania (2.3%) and 2 continent collaborations (2.3%). Of these included studies, there were 3 (6.3%) studies with a low risk of bias: Van Wessem et al. [84], Hardy et al. [94] and Kuorikoski et al. [96]. From the remaining, 53 (60.2%) were of moderate risk and 32 (36.4%) of high risk using ROBINS-I tool (Fig. 2).

Ten studies [29, 32, 43, 53, 61, 66, 91, 96, 103] defined polytrauma using the Berlin polytrauma definition (11.3%) and six studies [40, 62, 83, 88, 94, 97] used the Newcastle definition (6.8%). The remaining 72 studies defined polytrauma using other definitions: Seventy papers using ISS ≥ 16 (79.5%), one paper using ISS ≥ 17 [25] (1.1%) and one paper using the US Department of Veterans Affairs definition [92] (1.1%). Of the 10 studies using BPD, seven were studies from Europe and the other three were from Asia. For the Newcastle definition, three were studied in Asia, two in Oceania and one in Europe. For studies using ISS ≥ 16 to define polytrauma, 51 were from Europe, six from Asia, seven from North America and three from Africa. The other two papers using alternative definitions of ISS ≥ 17 [26] and the US Department of Veterans Affairs definition [92] were based in North America.

**Fig. 3:**
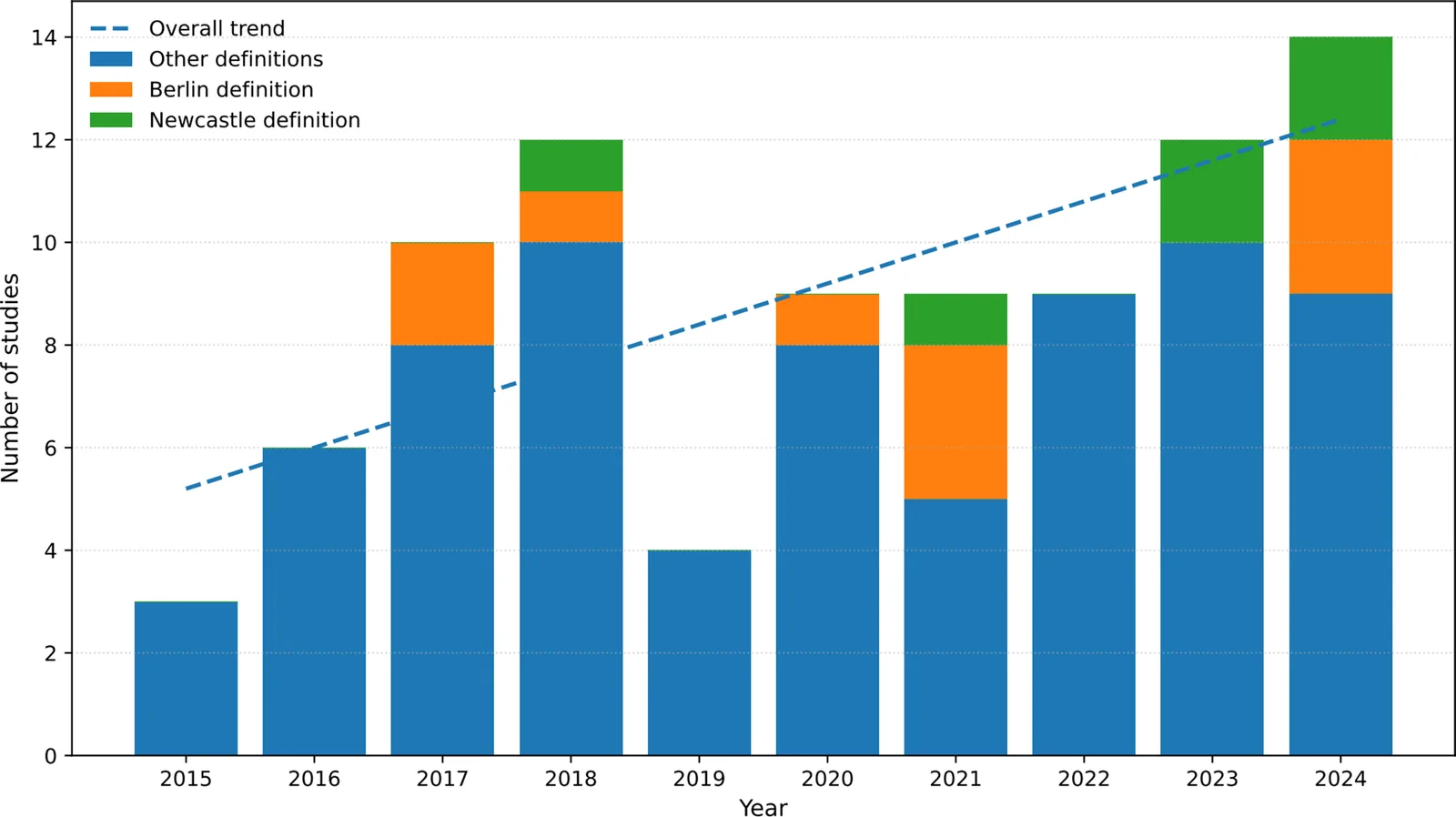
Definition of polytrauma reported by year with trendline

There was an increasing number of studies published focusing on the topic of polytrauma, with three studies identified in 2015, increasing to 14 in 2024. Figure 3 depicts the overall trend in increasing number of publications focusing on polytrauma. Figure 3 also outlines the usage of the Newcastle and the Berlin definitions of polytrauma by year. In 2024, these definitions were reported the most with two papers reporting the Newcastle definitions and three papers reporting the Berlin definition.

Overall, 3.7% (88 studies) of the included studies defined polytrauma explicitly and only 0.68% (16 studies) used the consensus definitions. The remaining 99.32% either provided no definition of polytrauma or used a non-objective definition, such as multi-system trauma.

**Table 1:** Summary of included studies by year and definition of polytrauma used

Author	Published Date	Study design	Location of study	Definition of polytrauma
Guisasola et al.[16]	2015	Prospective cohort	Spain	ISS ≥ 16
Stephan et al.[17]	2015	Retrospective cohort	Germany	ISS ≥ 16
Topp et al.[18]	2015	Retrospective cohort	Germany	ISS ≥ 16
Bedreag et al.[19]	2016	Retrospective single-centre	Romania	ISS ≥ 16
Ferree et al.[20]	2016	Prospective observational cohort	Netherlands	ISS ≥ 16
Kanezaki et al.[21]	2016	Retrospective cohort	Japan	ISS ≥ 16
Larsen et al.[22]	2016	Prospective cohort	Denmark	ISS ≥ 16
Van Der Eng et al.[23]	2016	Prospective cohort	Netherlands	ISS ≥ 16
Weinberg et al.[24]	2016	Retrospective cohort	United States	ISS ≥ 16
Arsenijevic et al.[25]	2017	Prospective cohort	Serbia	ISS ≥ 16
Dielwart et al.[26]	2017	Retrospective cohort	United States	ISS ≥ 17
El Mestoui et al.[27]	2017	Retrospective cohort	Netherlands	ISS ≥ 16
Ferree et al.[28]	2017	Prospective cohort	Netherlands	ISS ≥ 16
Frenzel et al.[29]	2017	Retrospective cohort	Austria	BPD
Lubelski et al.[30]	2017	Retrospective cohort	United States	ISS ≥ 16
Niedermeier et al.[31]	2017	Retrospective cohort	United States	ISS ≥ 16
Rau et al.[32]	2017	Retrospective cohort	Taiwan	BPD
Sapan et al.[33]	2017	Prospective cohort	Indonesia	ISS ≥ 16
Tosounidis et al.[34]	2017	Retrospective cohort	United Kingdom	ISS ≥ 16
De Vries et al.[35]	2018	Retrospective cohort	Netherlands	ISS ≥ 16
Dijkink et al.[36]	2018	Retrospective comparative cohort	Netherlands & United States	ISS ≥ 16
Frohlich et al.[37]	2018	Prospective cohort	Germany	ISS ≥ 16
Haider et al.[38]	2018	Prospective cohort	Austria	ISS ≥ 16
Halbgebauer et al.[39]	2018	Prospective cohort	Germany	ISS ≥ 16
Hsieh et al.[40]	2018	Cross-sectional cohort	Taiwan	Newcastle definition
Macke et al.[41]	2018	Retrospective cohort	Germany	ISS ≥ 16
Negrin et al.[42]	2018	Prospective cohort	Austria	ISS ≥ 16
Pothmann et al.[43]	2018	Retrospective cohort	Switzerland	BPD
Schicho et al.[44]	2018	Retrospective cohort	Germany	ISS ≥ 16
Van Der Vliet et al.[45]	2018	Prospective cohort	Netherlands & United States	ISS ≥ 16
Wiegner et al.[46]	2018	Prospective cohort	Germany	ISS ≥ 16
Bershadsky et al.[47]	2019	Prospective cohort	Russia	ISS ≥ 16
Liu et al.[50]	2019	Randomised controlled trial	China	ISS ≥ 16
Van Laarhoven et al.[49]	2019	Retrospective cohort	Netherlands	ISS ≥ 16
Winkelmann et al.[50]	2019	Retrospective cohort	Germany	ISS ≥ 16
Braunstein et al.[51]	2020	Prospective cohort	Germany	ISS ≥ 16
Dubrov et al.[52]	2020	Prospective cohort	Ukraine	ISS ≥ 16
Halvachizadeh et al.[53]	2020	Retrospective cohort	Germany & Switzerland	BPD
Jensen et al.[54]	2020	Retrospective cohort	Switzerland	ISS ≥ 16
Joubert et al.[55]	2020	Retrospective cohort	France	ISS ≥ 16
Schwartz et al.[56]	2020	Retrospective cohort	United States	ISS ≥ 16
Teuben et al.[57]	2020	Retrospective cohort	Germany & Switzerland	ISS ≥ 16
Wierzcholowski et al.[58]	2020	Retrospective cohort	Poland	ISS ≥ 16
Yeh et al.[59]	2020	Retrospective cohort	Taiwan	ISS ≥ 16
Briese et al.[60]	2021	Retrospective cohort	Germany	ISS ≥ 16
Driessen et al.[61]	2021	Retrospective cohort	Netherlands	BPD
Martins et al.[62]	2021	Cross-sectional cohort	Pakistan	Newcastle definition
Muhamedhussein et al.[63]	2021	Prospective cohort	Tanzania	ISS ≥ 16
Popal et al.[64]	2021	Retrospective cohort	Netherlands	ISS ≥ 16
Rugg et al.[65]	2021	Prospective cohort	Austria	ISS ≥ 16
Schmitt et al.[66]	2021	Retrospective cohort	Switzerland	BPD
Stolberg-Stolberg et al.[67]	2021	Retrospective cohort	Germany	ISS ≥ 16
Weihs et al.[68]	2021	Retrospective cohort	Austria	BPD
Ditsch et al.[69]	2022	Prospective cohort	Germany	ISS ≥ 16
Elmhiregh et al.[70]	2022	Retrospective cohort	Qatar	ISS ≥ 16
Khajehpour et al.[71]	2022	Prospective cohort	Iran	ISS ≥ 16
Mair et al.[72]	2022	Retrospective cohort	Germany	ISS ≥ 16
Slordal et al.[73]	2022	Retrospective cohort	Norway	ISS ≥ 16
Vicente et al.[74]	2022	Prospective cohort	United States & Canada	ISS ≥ 16
Vicente et al.[75]	2022	Prospective cohort	United States & Canada	ISS ≥ 16
Weber et al.[76]	2022	Prospective cohort	Germany	ISS ≥ 16
Zeelenberg et al.[77]	2022	Retrospective cohort	Netherlands	ISS ≥ 16
Becker et al.[78]	2023	Retrospective cohort	Germany	ISS ≥ 16
Belger et al.[79]	2023	Retrospective cohort	Germany	ISS ≥ 16
Biber et al.[80]	2023	Retrospective cohort	Germany	ISS ≥ 16
De Vries et al.[81]	2023	Cross-sectional cohort	Netherlands	ISS ≥ 16
Foster et al.[82]	2023	Randomised clinical trial	United States	ISS ≥ 16
Tan et al.[83]	2023	Retrospective cohort	Singapore	Newcastle definition
Van Wessem et al.[84]	2023	Prospective cohort	Netherlands	ISS ≥ 16
Weber et al.[85]	2023	Prospective cohort	Germany	ISS ≥ 16
Weber et al.[86]	2023	Prospective cohort	Germany	ISS ≥ 16
Weber et al.[87]	2023	Prospective cohort	Germany	ISS ≥ 16
Weihs et al.[88]	2023	Retrospective cohort	Austria	Newcastle definition
Wollner et al.[89]	2023	Retrospective cohort	Austria	ISS ≥ 16
Ahmed et al.[90]	2024	Prospective cohort	Egypt	ISS ≥ 16
Arslan et al.[91]	2024	Retrospective cohort	Turkey	BPD
Brostow et al.[92]	2024	Cross-sectional cohort	United States	At least 1 mild traumatic brain injury with at least 1 comorbidity (US Department of Veterans Affairs definition)
Dubivska et al.[93]	2024	Prospective cohort	Ukraine	ISS ≥ 16
Hardy et al.[94]	2024	Retrospective cohort	Australia	Newcastle definition
Jaekel et al.[95]	2024	Prospective cohort	Germany	ISS ≥ 16
Kuorikoski et al.[96]	2024	Retrospective cohort	Finland	BPD
Merakis et al.[97]	2024	Retrospective cohort	Australia	Newcastle definition
Ritter et al.[98]	2024	Prospective cohort	Germany	ISS ≥ 16
Schuster et al.[99]	2024	Retrospective cohort	Germany	ISS ≥ 16
Seidu et al.[100]	2024	Cross-sectional cohort	Ghana	ISS ≥ 16
Stormann et al.[101]	2024	Retrospective cohort	Germany, Austria, Switzerland	ISS ≥ 16
Weber et al.[102]	2024	Prospective cohort	Germany	ISS ≥ 16
Zhang et al.[103]	2024	Retrospective cohort	China	BPD

## Discussion & conclusion

As our current systematic review focused specifically on polytrauma, other forms of trauma beyond polytrauma, including severe isolated injuries, are difficult to comment on directly. However, existing evidence indicates that polytrauma-defined patients have significant worse outcomes than major trauma patients with a comparable injury severity score [10], reinforcing the rationale for maintaining polytrauma as a distinct, precisely defined entity rather than treating it as interchangeable with major trauma more broadly.

Polytrauma is associated with severe injury of multiple body regions and pathophysiological changes, due to transfer of large kinetic energy affecting various systems. Despite being more than 10 years since consensus, numerous definitions of polytrauma targeting different populations are still in use, with the ISS threshold of ≥ 16 remaining the most used definition of major trauma, based on anatomically coded injuries only.

The ISS threshold of ≥ 16 is one of the frequently employed definitions of major trauma and it is frequently used synonymously with polytrauma. This definition includes severe monotrauma or multiply injured patients with one severe injury and some other minor injuries not worsening mutually each other like in polytrauma [4].

The Newcastle and the Berlin Definition of polytrauma both satisfy the anatomic criteria by including patients with at least two affected AIS body regions, which would include higher risk patients with higher mortality and compromised prognosis [10].

The key difference between the two consensus definitions is that the Newcastle definition is purely anatomical score based (AIS), but with the AIS > 2 in at least two body regions threshold, some physiological compromise and systemic inflammation always exist, without the need for specific coding. This allows wider use around the world as most registries do not have comprehensive physiological data to code polytrauma according to the Berlin definition. The use of multiple physiological confounders in the Berlin definition can result in a much higher mortality cohort, but the subgroup of patients with 1 physiological confounder has the same mortality rate (14%) as the cohorts defined by the Newcastle definition [4]. Not surprisingly, the addition of further physiological confounders to the anatomical definition, leads to a stepwise higher mortality rate and less patients included with more confounders [104]. The mortality rate was 2.9% when no physiological confounder was present, 13.9% when one parameter is met and up to 89.3% with all five parameters.

A recent retrospective cohort study by Driessen et al. [13] assessed the Berlin polytrauma definition using the Dutch trauma registry data and compared its results to the Deutcsche Gesellschaft für Unfallchirurgie Trauma Register (DGU-TR) [12], which was the registry used to develop the Berlin polytrauma definition. The study concluded that only 1.4% (4,264 from 300,649 total patients recorded) met the Berlin polytrauma criteria, however, provided similar results to when the definition was applied to DGU-TR. It confirmed that the Berlin polytrauma definition aids in identification of severely injured patients with high risk of mortality (27.2%), intensive care unit admission (71.2%) and hospital length of stay (10 days). These findings are not surprising as the Berlin definition was aimed to identify a population with approximately 30% mortality based on the DGU-TR.

The main limitation of the Berlin definition is that the physiological parameters required to correctly identify its representing population, are not readily reported in every trauma registry, while AIS is reported in all. Furthermore, the physiological confounders can be related and likely to be met in a nonlinear fashion such as hypotension, acidosis and coagulopathy, which are all linked to severe bleeding and consequent traumatic shock. Another point to note is that initial low GCS (unless it is coupled with head injury AIS) does not necessary define traumatic brain injury, as it can be due to medication used for extraction, severe hypoperfusion or intoxication.

Interestingly, only 38.5% of the polytrauma patients based on Newcastle definition did not have any additional confounders and the Newcastle definition based polytrauma mortality is 14%, which is exactly the Berlin definition based polytrauma mortality with one physiological confounder [12]. This highlights the very good performance of the Newcastle definition even without recording any physiological parameters to define polytrauma from large registries and from resource limited locations.

Polytrauma is an immense burden on society, as it accounts for almost 50% of traumatic fatalities and the survivors being left with chronic complications, ultimately highlighting the importance of targeted comprehensive care and accurate patient identification [105]. Thirty years ago, the polytrauma population represented by the ISS threshold of greater than 15 points, had an expected mortality rate of greater than 20% [8]. However, with enhancements in trauma care and management, the same population has a significantly lower mortality rate of 9% to 12.3% [15]. Since the initial recognition of requiring consensus for the definition of polytrauma 16 years ago [106], there has been notable advancements in recognising polytrauma as a disease.

The mechanism, pathophysiology, symptomatology, management and complications of polytrauma are well established. It is due to high mechanical energy transfer to the body resulting in the activation of mechanoreceptors and toll-like receptors, triggering the body’s acute innate immune system and disrupting homeostasis. As a result, the signs/symptoms of altered GCS, hypothermia, tachycardia, hypotension and other alterations in homeostatic functions occur. Consequently, the secondary complications of coagulopathy, sepsis, organ failure and catabolism syndrome may occur. The current mainstay management comprises of initial pre-hospital optimisation, comprehensive diagnostic work-up followed by symptom/sign specific resuscitation and continued follow up for prevention of secondary complications [103].

As the definition and identification of polytrauma can be philosophical, some specialists regard polytrauma as a list of injuries, rather than a disease entity on its own. A recent retrospective cohort study published in 2024 by Zhang et al. [103] concluded that polytrauma is not a series of isolated events, but rather a public health burden with an associated high rate (44.6%) of functional impairment from previous trauma requiring hospital care within the past 5 years (trauma phenomenon, known as recidivism). Hence further underscoring the importance of precisely defining polytrauma for further implementation of prevention measures and maximisation of patient outcomes. Fundamentally, polytrauma can fulfill the definition of disease, whether it be acute or chronic.

This systematic review highlighted the need for further education and advocacy for the accurate definition of polytrauma, which will be a code-able disease condition in the World Health Organization’s International Classification of Diseases 11th edition (WHO ICD-11). Although this study was limited to a systematic review and did not consider meta-analysis focusing on the outcome of in-hospital mortality, further studies exploring this would be helpful in solidifying the status of polytrauma as a disease entity.

## Data Availability

No datasets were generated or analysed during the current study.
